# The Perspective of DMPK on Recombinant Adeno-Associated Virus-Based Gene Therapy: Past Learning, Current Support, and Future Contribution

**DOI:** 10.1208/s12248-021-00678-7

**Published:** 2022-01-31

**Authors:** Nancy Chen, Kefeng Sun, Nagendra Venkata Chemuturi, Hyelim Cho, Cindy Q. Xia

**Affiliations:** Takeda Development Center Americas, Inc. (TDCA), 35 Landsdowne Street, Cambridge, Massachusetts 02139 USA

**Keywords:** biodistribution, first-in-human (FIH), gene therapy, immunogenicity, recombinant adeno-associated virus (rAAV)

## Abstract

Given the recent success of gene therapy modalities and the growing number of cell and gene-based therapies in clinical development across many different therapeutic areas, it is evident that this evolving field holds great promise for the unmet medical needs of patients. The recent approvals of Luxturna® and Zolgensma® prove that recombinant adeno-associated virus (rAAV)-based gene therapy is a transformative modality that enables curative treatment for genetic disorders. Over the last decade, Takeda has accumulated significant experience with rAAV-based gene therapies, especially in the early stage of development. In this review, based on the learnings from Takeda and publicly available information, we aim to provide a guiding perspective on Drug Metabolism and Pharmacokinetics (DMPK) substantial role in advancing therapeutic gene therapy modalities from nonclinical research to clinical development, in particular the characterization of gene therapy product biodistribution, elimination (shedding), immunogenicity assessment, multiple platform bioanalytical assays, and first-in-human (FIH) dose projection strategies.

**Figure:**
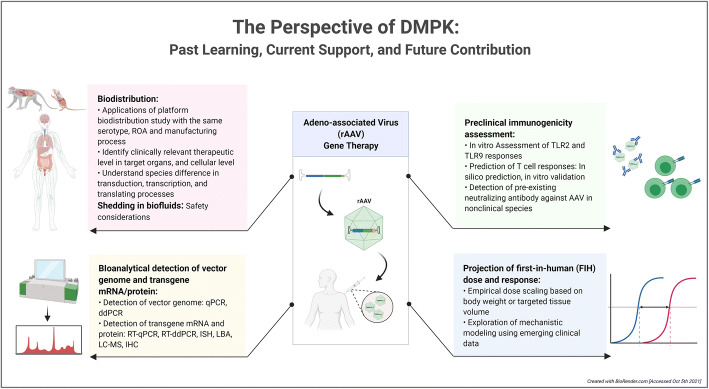
Graphical abstract

Graphical abstract

## Introduction

Given the recent success of recombinant adeno-associated virus (rAAV) vectors (i.e., Zolgensma® for spinal muscular atrophy and Luxturna® for hereditary blindness), a growing number of cell- and gene-based therapies are in clinical development across many different therapeutic areas (1). These disease-modifying therapies can be a transformative and curative treatment option for patients with genetic diseases that progress rapidly to fatal conditions with limited or no therapeutic options. Gene therapy is typically categorized into *ex vivo* and *in vivo*; the latter is further sub-categorized into viral and non-viral delivery. The focus of this review paper is on the *in vivo* rAAV vector gene delivery. Naturally occurring AAV is a replication-defective virus with a linear single-stranded DNA (ssDNA) genome, and it infects humans and primates. Although devoid of the replication (*rep*) and capsid (*cap*) sequences, rAAV can infect both dividing and non-dividing cells and persist primarily in episomal DNA intracellularly (2). Despite the nonclinical and clinical successes, the development of rAAV-based gene therapy is still associated with many challenges, such as limited persistence of transgene expression and immune responses to both the vector and transgene products. Although multiple guidelines from regulatory agencies on gene therapy have been issued in the last two years (3–6), the practical details and scientific approaches on rAAV from an industry drug development perspective to meet regulatory requirements are minimally described. Since the rAAV-based gene therapy field is evolving, it will benefit from an industry perspective especially when determining species-specific differences in biodistribution, quantitative pharmacokinetic (PK)/pharmacodynamic (PD) relationships, and FIH dose projections. Improving our understanding on these critical components in the discovery, development, and clinical translation of rAAV-based gene therapy can potentially expedite the path to clinical success.

## Concepts of Pharmacokinetics (PK)/Pharmacodynamics (PD) for rAAV-BASED GENE THERAPY

**Fig. 1:**
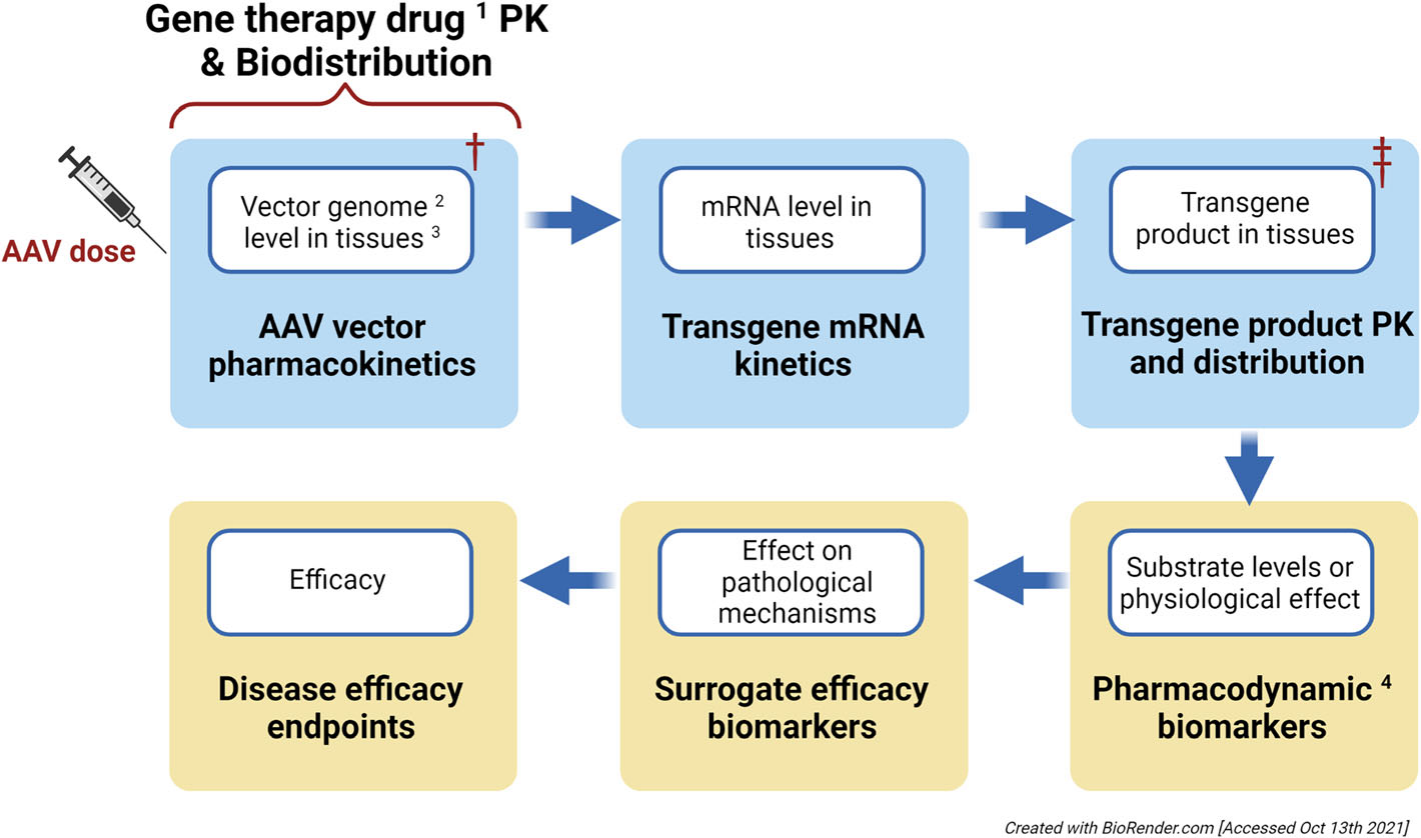
Flow chart of PK/PD concept for rAAV-based gene therapy. ^1^ Drug = Vector = Capsid + vector genome; ^2^ Vector genome is used as a surrogate of GT drug product; ^3^ Tissues include plasma and solid organs; ^4^ PD includes target engagement and/or pathway modulation. Diagram is consistent with Expectations for Biodistribution (BD) Assessments for Gene Therapy (GT) Products, International Pharmaceutical Regulators Programme (IPRP) 2018. † Can be affected by pre-existing anti-AAV antibodies, anti-capsid and/or anti-transgene product T cell response, and other stresses. ǂ Can be affected by anti-transgene product T cell response, anti-transgene product neutralizing antibodies, and other stresses

The concept of PK and PD for rAAV-based gene therapy is distinct from other therapeutic modalities and the conventional absorption, distribution, metabolism, and elimination (ADME). Hence, typical PK/PD concepts do not adequately describe the physiological processes that apply to rAAV-based gene therapy. The PK of the rAAV-based gene therapy drug product only refers to the disposition of rAAV (capsid and DNA vector genome) and is more commonly referred to as “biodistribution” (7). For rAAV-based gene therapy, the nominal PK portion can be separated into three components: the AAV vector genome, transgene mRNA, and transgene product. The vector genome is the quantifiable component of rAAV-based gene therapy product, and it is often used as a surrogate for the kinetics of the gene therapy product because of the lack of reliable methodology in quantifying the viral capsid in tissues and organs (8, 9). After capsid uncoating and the formation of episomal viral DNA, the transgene mRNA and the transgene product can be formed in both target and off-target tissues (Figure 1). The transgene protein level is the most relevant PK measurement because it is perceived as the traditional exposure level that drives the PD biomarkers. Therefore, the transgene product is often the focus of PK analysis in addition to the vector genome. The kinetics of transgene mRNA are assessed in the absence of feasible bioanalytical methods to quantify the transgene product. They are also assessed when a new promoter/enhancer, an engineered transgene sequence, and/or regulator elements are incorporated into the vector design, or when cross-species comparison in transcriptional activity is needed. The PD components are measures of physiological responses, pathological modification, and efficacy endpoints, and are connected to transgene product PK as depicted in Figure 1.

The durability and persistence of the transgene product as well as the clinical benefits are crucial aspects for therapeutic evaluations of gene therapy. According to regulatory guidance (3, 4, 6), durability of both the vector DNA and the transgene product (protein) should be investigated in pharmacological and toxicological animal models. Regardless of the target tissues, the characterization of biodistribution should consist of vector genome spreading from the site of administration to the transduced tissues (distribution), vector genome duration in tissues (persistence), and vector genome elimination from the tissue (clearance). On the other hand, the definition of distribution, persistence, and clearance of the transgene product can be applied differently based on its bio-production and target sites. Table I describes three possible scenarios of where the target site is in relation to the transduced (bio-production) organs. For non-secreted transgene proteins, the protein is measured from the same transduced tissues from which viral genomes are also assayed. In this case, there is no need to measure the transgene protein in blood, and the vector genome detected in blood is considered a marker of shedding (elimination from tissues). Another commonly used approach is cross-correction, wherein the transgene protein is produced and secreted out of transduced tissue(s) into circulation and then is expected to be taken up by target organ(s). In this case, it is critical to assess the cross-corrected tissues and biofluids, such as blood and CSF, at multiple time points in the context of PK/PD correlation in the pharmacology model. Protein cross-correction is defined as when the functional protein (including proper enzymatic activity) is taken up by other tissues or cells after being secreted from the bio-production organ(s). For transgene products that function in systemic circulation (e.g., Factor VIII [FVIII], Factor IX [FIX]), their distribution and persistence are primarily characterized using the serum/plasma, although distribution in off-target tissues is also frequently assessed to address potential safety concerns. For the latter two scenarios, the protein distribution in non-production organs may be estimated by physiologically based protein distribution from serum/plasma protein PK (10).

**Table I:** Definition of Distribution, Persistence, and Clearance for Transgene Product (Protein)

Transgene product (protein)^#^	Distribution - protein expression in tissues as a function of translation, degradation, and transport to other tissues if secreted	Persistence – how long the protein is present in relevant tissues	Clearance - protein removal from tissues
Scenario 1	Protein remains within transduced tissue cells where it functions (ideally target tissue only).		Degradation (lysosomal or proteasomal) within transduced cells
Scenario 2	Protein is secreted out of the transduced tissue cells into systemic circulation for cross-correction through uptake by other tissues where it functions (ideally cross-corrected target tissues only).		1) Secretion from the transduced tissues 2) Elimination from the systemic circulation through host immune system and renal and hepatic clearances 3) Degradation within transduced and cross-corrected (or off-target) tissues that uptake protein form circulation
Scenario 3	Protein is secreted out of the transduced tissue cells into systemic circulation where it functions. Protein may be passively taken up by other tissues.		

^**#**^Transgene product could also be miRNA; only scenario of expression within transduced cells applies

## Assessment of Biodistribution and Shedding of Gene Therapy Drug Product and PK/Distribution of Transgene Product

Understanding the biodistribution of a gene therapy product in nonclinical species is critical for establishing its safety and efficacy prior to clinical development. The quantitative biodistribution pattern of an rAAV-based modality is influenced by its serotype, route of administration (ROA), the animal species (including characteristics like age, sex, and strains), the manufacturing process, and bioanalytical techniques used in the study. The biodistribution characterization approach of rAAV may vary at different stages of the gene therapy program. During lead generation, the DMPK function may conduct a platform biodistribution study that is suitable for early discovery stages of an rAAV-based therapy before finalizing the vector design. If the sponsor decides to engineer the capsid to improve tissue tropism, or to modify the cassette sequence, a new biodistribution study with the final gene therapy product intended for clinical trials will need to be conducted. However, since regulatory agencies recognize that a high number of animals are necessary for a proper biodistribution assessment, in some instances, they have suggested alternatives such as conducting *in vitro* studies and/or *in silico* modeling (4, 11). In addition, when the same capsid serotype is used as a platform capsid to treat multiple indications by the same ROA and vector process development, the biodistribution data generated in an appropriate transducible nonclinical species (non-disease model, e.g., non-human primates) can inform and support multiple programs. Leveraging the established platform data may allow for a limited or abbreviated biodistribution assessment to be sufficient for the pivotal investigational new drug application (IND)-enabling Good Laboratory Practice (GLP)-compliant toxicology studies, which would be more cost effective. An abbreviated biodistribution assessment reduces animal usage, optimizes and streamlines manufacturing processes (as the capsid remains the same), saves resources, and reduces the time dedicated to manufacturing. According to FDA guidelines, such approaches or strategies that reduce animal usage can be considered, but should be communicated to the FDA well in advance of the IND filing, perhaps in an INTERACT meeting, for the sponsor to gain alignment with the agency (4, 11).

In general, the dose range, ROA, targeted patient population, manufacturing process, and bioanalytical setup of a definitive nonclinical biodistribution study for rAAV-based gene therapy should mirror as much as possible the intended clinical settings. In particular, the ROA is an important aspect of consideration in biodistribution study design. In drug discovery stages, where the ROA is often still being determined as the sponsor optimizes the vector biodistribution for different target tissues, a comparison of the biodistribution profiles among different ROAs is warranted. The tissue distribution should be considered even after local administration such as intra-articular (12) and intra-cisterna magna (ICM) delivery, since rAAV can readily circulate into systemic blood and other biofluids as well as to peripheral organs (13, 14).

At Takeda, our experimental experiences have demonstrated that in addition to characterizing vector biodistribution, it is often critical to thoroughly characterize the distribution and persistence of mRNA and/or transgene protein in the same study. However, the rAAV PK or biodistribution could be sequentially and mechanistically linked to downstream effects in the same experimental setting (Figure 1). Optimization of the promoter and enhancer in the expression cassette is another factor driving the strategy of assessing key tissues for the investigated disease. If the liver is used as the sole bio-production organ to secrete the transgene protein into systemic circulation for cross-correction of other tissues (and depending on the AAV dose, transduction efficiency, and the therapeutic target level of protein in non-liver tissues), it may be challenging for protein uptake in resistant tissues to achieve sufficient levels of the transgene protein and to maintain the protein level above a therapeutic threshold for multiple years. As an alternative to liver-mediated cross-correction, either a ubiquitous promoter or tissue-specific promoter may be used (e.g., a neuronal or muscular promoter) to drive localized translation in target tissues. For the ubiquitous promoter approach, assessment of vector genomes, transgene mRNA, and transgene protein in target tissues is needed to ascertain sufficient localized transduction and downstream protein production, which reduces reliance on the cross-correction mechanism.

One of the safety considerations for a gene therapy product is viral shedding in biofluids (6, 15). Although shedding data in animal studies is not required by all regulatory agencies such as by the FDA guidance (15), sponsors often still assess viral shedding because global submissions are usually intended. The duration of the viral shedding evaluation is usually the length of the study, with the aim of detecting a low or insignificant level of virus at the end of the study period. Viral shedding in the biofluid and feces is usually assessed in both IV and local administration in nonclinical species in a GLP-compliant toxicology study to ensure the safety of both the environment and the caretakers. The assessed biofluids usually include the whole blood, urine, feces, and sometimes the saliva. For ICM or subretinal injection routes, CSF and ocular fluids (e.g., tear), respectively, are also recommended for evaluation.

Biodistribution studies beyond characterization of vector shedding are not routinely performed on human subjects during clinical trials. This dearth of characterization studies poses a major challenge to understanding how findings in nonclinical species translate to the clinic. For tissues such as the liver and muscles where established safe biopsy procedures are available, vector genomes as well as transgene mRNA and protein can be analyzed from biopsied samples. Currently, quantitative and qualitative information on distribution and persistence of AAV vector DNA, mRNA, and transgene protein in somatic tissues in human is available for onasemnogene abeparvovec (Zolgensma®; vector DNA, mRNA, and SMN protein in multiple tissues), AAV5-hPBGD (vector DNA and mRNA in liver), and valoctocogene roxaparvovec (Roctavian®; vector DNA, mRNA, and FVIII in liver) (Table II) (16). These results are consistent with general patterns observed in nonclinical biodistribution studies for IV-administered AAV9 (17, 18) or AAV5-based modalities (19, 20). However, an exact comparison between humans and the nonclinical species may not be feasible because of the sample size and difference in collection time points. Nevertheless, because of ethical concerns and the limited dataset available for humans (Table II), it remains difficult to make definitive comparisons about the biodistribution profile of a specific AAV serotype between species.

**Table II:** Comparison of Biodistribution of AAV-Based Modalities Between Nonclinical Species and Human

Modality (capsid used)	Biodistribution in nonclinical species and human, with dose, age at vector administration, and collection time post dose		
	Mouse	Non-human primates	Human
Onasemnogene abeparvovec (AAV9) (18, 69, 70)	2.37×10^14^ vg/kg, neonatal FVB/NJ mice (M&F), week 12: Heart, 1.69×10^5^vg/μg DNA Lung, 3.56×10^4^vg/μg DNA Liver, 2.47×10^4^vg/μg DNA	N/A	1.1×10^14^ vg/kg, pediatric patients, 1.7 or 5.7 months: Highest in liver, followed by spleen, heart, inguinal lymph node, and other organs
AAV5-hPBGD (71, 72)	N/A	5×10^13^ vg/kg, cynomolgus monkeys (age 4, M&F), day 30: Adrenal gland: 6.10×10^5^vg/μg DNA Spleen: 5.52×10^5^vg/μg DNA Liver: 3.56×10^5^vg/μg DNA	6×10^12^ vg/kg, adult, 1 year: Liver: 1.43×10^4^vg/μg DNA 1.8×10^13^ vg/kg, adult, 1 year: Liver: 2.24×10^3^vg/μg DNA
Valoctocogene roxaparvovec (AAV5) (16, 19, 20)	6×10^13^ vg/kg, FVIII/RAG2 double knockout mice (8-9 weeks old, M), day 91: Liver, 1.7 vg/diploid genome	6×10^13^ vg/kg, cynomolgus monkeys (age 2.5-4.4, M&F), day 56: Liver: 7.5 vg/diploid genome	6×10^12^ vg/kg, adult, 3.9 years: Liver: 0.1 vg/diploid genome 4×10^13^ vg/kg, adult, 2.7 years: Liver: 1.7 vg/diploid genome

Only mean levels in the top three or four organs ranked by detected vector genome levels are listed. Quantitative human biodistribution data for onasemnogene abeparvovec have not been formally published. *PBGD*, porphobilinogen deaminase; *M*, male; *F*, female; *N/A*, data not available

## Bioanalytical Support for Biodistribution and Viral Shedding

In nonclinical studies, there are many potential analytes that need diverse analytical platforms. In this review, the methods used for the determinations of vector genome, transgene mRNA, and the transgene product (protein) (Table III) as well as immunogenicity assays for rAAV-based gene therapy products (Table IV) are briefly summarized. The analytical method details can be found in other published literature reviews (21–28).

**Table III:** Bioanalytical Methods to Understand the Biodistribution of Gene Therapy Product and Transgene Product

Analyte	Objectives	Analytical method	Assay type
Vector or Transgene DNA	• Understand biodistribution and shedding of GT product • Correlate with efficacy and safety	qPCR ddPCR	• Quantitative measurement of total amount. • ddPCR provides an absolute quantification of copies*/*mL with a smaller dynamic range and limited multiplexing capacity.
		ISH	Semi-quantitative measurement with cellular location.
Transgene mRNA	• Confirm transgene transcription • Correlate with efficacy and safety	RT-qPCR ddPCR	Quantitative measurement of total amount.
		ISH	Semi-quantitative measurement with cellular location.
Transgene Protein	• Confirm transgene expression • Correlate with efficacy or any tissue-specific detrimental effects • Serve as biomarker in some cases • Understand therapeutic durability • Establish PK/PD for dose selection	LBA LC/MS	• Quantitative measurement of total protein • Advantage of LC/MS: - Allows for distinguishing the human transgene protein from the endogenous protein in a nonclinical species. - Characterizes various engineered proteins, such as the shortened, truncated transgene proteins, compared with the endogenous form. - Enables high and reproducible recovery, especially for transgene protein quantification in tissues. - Reduces the assay variability by using an internal standard (IS) like an isotopically labeled peptide, a flanged peptide (with a tryptic cleavage site) or a full-length protein (24, 35).
		Flow cytometry	Quantitative measurement and differentiation of surface and intracellular proteins.
		Western blot	Semi-quantitative measurement of intact or truncated protein and alternative replaced by LC/MS.
		IHC imaging	Semi-quantitative measurement with cellular location.

**Table IV:** Immunogenicity Evaluation for Gene Therapy in Nonclinical Animal Models

Immune response	Measurement	Objectives	Rationale	Methodology
Pre-existing humoral immune response	Anti-capsid neutralizing antibody	Group or exclude the animals from studies Data interpretation: • Post-dose magnitude of immune response may relate to adverse effects.	• Local injection is immune privileged from immunogenicity. There is NO need to exclude AAV NAb + animals in bio/distribution studies. • IV injection needs to exclude AAV NAb + animals to avoid negative impact on transduction efficiency.	Cell-based assay
Adaptive humoral immune response	Anti-capsid total antibody (TAb) Anti-transgene product antibody (TAb)	• The ADA for transgene product may impact the PK and the persistence of therapeutic protein.	• Any systemic exposure of the viral vector for a couple of days or more can potentially prompt anti-capsid ADA. • Similarly, local injection can have enough capsid exposure in the CSF and/or serum to trigger immune response. • rAAV capsid triggers innate immune response. • The transgene product can elicit a humoral immune response to generate transgene product-specific antibodies that can compromise therapeutic efficacy.	ELISA, MSD, other LBA
Adaptive humoral immune response	Anti-transgene product neutralizing antibody	Data interpretation: Antibodies may impact the PK and biological activity of transgene product.	• NAb by transgene product is usually not needed in nonclinical space. Either a pharmacology biomarker or enzyme activity in serum can be used as a surrogate for anti-transgene product NAb. • Can consider for internal knowledge learning purpose.	Cell-based, enzyme activity assays
Adaptive cellular immune response	Anti-capsid T cell response Anti-transgene T cell response	Data interpretation: Post-dose cellular immune response may relate to adverse effects or reduced expression	Will be triggered with pharmacological or toxicological findings.	ELISPOT, CTL assays, flow cytometry (PBMC or tissues such as liver and spleen)
Cytokine secretion is associated with innate immune reaction to viral coat and nucleic acid	Cytokine quantification (IFNγ, TNFα, IL-1β, IL-6)	Assays will be conducted if GTx has been associated with cytokine-relatedsafety concerns	Will be triggered with pharmacological or toxicological findings.	LBA, especially multiplexed (Luminex, MSD, Quanterix)
Complement activation	Complement factors (C3, C4 C3a desArg)	Assays will be conducted if GTx has been associated with AEs (thrombocytopenia, hepatotoxicity)	Will be triggered with pharmacological or toxicological findings.	LBA, serum protein analyzers

### Detection of Vector Genome

Quantification of nucleic acid sequences of rAAV vector DNA is important in assessing the relative biodistribution of a gene therapy product and in determining the kinetics of its accumulation and decay in tissues/biofluids, or virus shedding in excreta. The FDA recommends a quantitative, sensitive assay such as quantitative PCR (qPCR), to analyze the samples for vector genome sequences (11). In the absence of regulatory guidance for PCR assay validation, it is suggested to follow a scientific approach for method development and validation strategies, with the support from the Minimum Information for the Publication of Quantitative Real-Time PCR Experiments (MIQE) guidelines (29, 30). The MIQE guidelines focus on the consistency of qPCR performance and provide methodology considerations for the design of real-time or digital qPCR assays and experiments.

The most common form of digital PCR, droplet digital PCR (ddPCR), is a relatively new technology that utilizes microfluidics to partition target DNA into droplets where individual PCR reactions occur (31). Compared with the traditional real-time qPCR (TaqMan probe-based), ddPCR has advantages in that the technology provides an absolute quantification of copies*/*mL without the use of a standard curve, is less affected by matrix and sample inhibitors, and is considered more sensitive, precise, accurate, and reproducible (32). However, ddPCR is generally more expensive, has a smaller dynamic range, and has limited multiplexing capacity. Until recently, qPCR was the standard for quantifying genomic material, but in the future, we anticipate more applications of ddPCR in quantifying vector DNA and transgene mRNA in gene therapy because of its advantages over traditional qPCR, and the knowledge, experience, and improvement of that is been amassed for this new technology (21, 22).

### Detection of Transgene mRNA and Protein

Determination of the transgene mRNA and transgene protein profile in target and non-target tissues in nonclinical species can assist in gene construct selection, dose recommendation for nonclinical efficacy and safety studies, and initial clinical dose selections. There are several classes of transgene proteins that require detection. These include soluble proteins, enzymes, and structural and membrane proteins as well as intracellular proteins located in specific subcellular compartments. Depending on the class of the protein, a variety of technologies, such as ligand binding assays (LBA), Western blots, tissue staining, protein liquid chromatography–mass spectrometry (LC–MS), and flow cytometry, can be applied for transgene protein detection or quantification (33, 34). In recent years, protein LC–MS, particularly when combined with immune-affinity (IA) enrichment, has emerged as an attractive and alternative platform for sensitive and selective protein biomarkers and protein therapeutic quantification (35). A combination of existing guidelines for chromatography assays (for small molecules) and LBA assays (for large molecules) is used for the validation of hybrid LBA/LC–MS methods (36).

When there is a lack of reagent to purify or detect the transgene product or when it is difficult to differentiate the human protein from the endogenous protein in animal models, assessment of transgene mRNA is critical to understand the transduction efficiency, optimize the gene construct, and build a dose/efficacy or dose/safety relationship. Transgene mRNA can be quantitatively measured by reverse transcription PCR (RT-qPCR). Transgene mRNA can be expressed as either absolute (copy numbers of mRNA in a given sample, presented as copies of mRNA per μg total RNA) or relative quantification (gene expression of a particular gene of interest in treated samples relative to the level of gene expression in an untreated sample, presented as 2^ΔΔCT^) (37). The advantage of relative quantification is that it does not require the generation of a standard curve. However, to establish the quantitative relationship of transcription to either translation or transduction in a per target cell basis, the absolute quantification of mRNA is typically used.

### Morphology-Based Biodistribution

While qPCR and RT-PCR provide accurate quantitation of vector DNA and transgene mRNA, respectively, these assays lack the cellular context necessary for an assessment at the level of a single cell. Morphology-based biodistribution complements amplification-based techniques by adding spatial context to the assessment within a tissue. Among others, fluorescent reporter (imaging study) or immuno-histochemistry (IHC) for reporter protein or tagged transgene protein, and *in situ* hybridization (ISH) for DNA (38) and mRNA are examples of morphology-based biodistribution. Such cellular distribution may provide insights that can influence pharmacology and safety considerations of the expression of a given transgene in a cell type with a unique physiological function (39).

## Nonclinical Immunogenicity Assessment

Nonclinical and clinical studies have shown that rAAV-based gene therapy can elicit host immune responses (innate and adaptive) and that pre-existing immunity to AAV vector can interfere with therapeutic efficacy (40). In order to develop a strategy to optimize the vector and transgene designs and to optimally manage immunogenicity, it is critical to understand the complex relationship between rAAV vectors and the host immune response in the nonclinical space. This understanding can then be used to develop relevant nonclinical models for immunogenicity risk assessment and understand the predictability of these models on clinical safety and efficacy. Pre-existing and post-treatment neutralizing antibodies (NAb) against AAV capsid have been shown to decrease the rAAV-transduction efficiency (40). At the cellular level, the rAAV capsid and the CpG motif of a genome can be recognized by toll-like receptor (TLR)2- and TLR9-mediated innate immune pathways, respectively (41). Capsid peptides presented by major histocompatibility complex (MHC) I (CD8+ T cell activation) and MHC II (CD4+ T cell activation) can induce cellular and humoral immunogenicity, thus limiting the ability to re-dose with rAAV-based gene therapy products (40). Taken together, all of these factors present major challenges to rAAV-based gene therapies.

Over time, an integrative and multi-tiered approach to evaluating immunogenicity to both transgene and capsid of rAAV has emerged within the industry (42, 43). In general, the series of assay platforms to evaluate immunogenicity that we typically consider (i.e., *in silico* prediction*, in vitro* cellular assays, LBA*, ex vivo* studies combined with nonclinical species [e.g., NHP] *in vivo* testing) can be applied during the early vector optimization and candidate selection phases in order to predict innate and adaptive immune responses. The selection of a particular assay platform and readout should be based on the immunogenic component being assessed (44). To capture the overall inflammatory signature in response to rAAV treatment, in which the secretion of pro-inflammatory cytokines including interleukin (IL)-6, tumor necrosis factor (TNF)-α, and type I/II interferon (IFN) was measured, the human peripheral blood mononuclear cell (hPBMC) assay has been developed. The activation of both the TLR2 and the TLR9 pathway can be inferred from the secreted levels of these effector molecules (pro-inflammatory cytokines) (45, 46). A reporter cell line expressing human TLR2- or TLR9-activation-dependent response element can be used as a mid- or high-throughput screening based on the immunogenic properties of CpG levels within its vector genome or its rAAV capsid. Assessment of the production of pro-inflammatory cytokines and IFN is also important as they can activate a pattern recognition receptor (PRR) pathway that leads to increased immune cell (e.g., neutrophil, macrophage, and dendritic cell) recruitment. Such immune cell recruitment can range from innate to adaptive immunity and plays a significant role in T cell priming for adaptive immunity induction (47).

T cell response is also considered one of the critical components to evaluate the rAAV platform during lead optimization (44). Our internal *in silico* tool utilizes the immune epitope database and analysis resource (IEDB) (48) and an internally developed machine learning algorithm to predict potential T cell epitope binding strength to either MHC I or II (49). The IEDB contains the largest global collection of experimentally measured immune epitope binding data for reference sets of MHC I and II alleles. Based on IEDB guidelines, a percentile rank is generated by comparing the peptide’s predicted IC_50_ against those of a set of random peptides from the SWISS-PROT database (48). Epitopes that are predicted to have higher binding affinity are more likely to be displayed in the context of MHC molecules, which then can be recognized by their corresponding T cell receptor (TCR) leading to T cell activation (cytotoxic T cell activation and anti-drug antibody [ADA]-producing B cell activation). In order to validate the *in silico* prediction and confirm T cell activation, both CD4+ and CD8+ T cell responses can be measured either *in vitro* using PBMC-based cellular assays or *ex vivo* using PBMC samples. We developed an enzyme-linked immune absorbent spot (ELISPOT)- and flow cytometry-based assay to detect the proliferation and activation of CD4+ and CD8+ T cell responses with further qualitative immunophenotyping to identify responsive subsets of T cells (i.e., effector memory T cells [T_em_], effector T cells [T_eff_], central memory T cells [T_cm_], and effector memory re-expressing CD45RA T cells [T_emra_]).

From these integrative and multi-tiered approaches, the ultimate result during the optimization of rAAV design (e.g., vector selection, capsid modification, promoter selection during vector optimization stage) and the *in vivo* assessment in nonclinical models is to mitigate the immunogenicity risk in clinical settings through more effective immunomodulation or immune ablation approaches.

### Detection of Anti-AAV Therapeutic Immunity in Nonclinical Species

Although there are no regulatory guidelines for immunogenicity assays in nonclinical studies, some concepts that are relevant to the design of ADA studies for nonclinical testing can be found in the International Council for Harmonisation (ICH) guidance for industry *S6(R1)* (50). This guideline recommends that antibody responses should be characterized (e.g., titer, number of responding animals, neutralizing or non-neutralizing) and their appearance should be correlated to the effects of antibody formation on PK/PD parameters, incidence and/or severity of adverse effects, complement activation, or the emergence of new toxic effects while interpreting the data. The evaluation of possible pathological changes related to immune complex formation and deposition should be evaluated. In most cases, the immune response in nonclinical species to biopharmaceuticals is variable, similar to the observations in humans. If interpretation of the data from a safety study is not compromised by these issues, then no special significance should be ascribed to the antibody and cellular immune response (50).

Currently, no appropriate animal model can predict immunogenicity of rAAV in humans (51, 52). However, animal models that are primarily used for pharmacology, biodistribution, and toxicology studies are important to understand the extent of impact of immunogenicity on efficacy and safety. Nonhuman primates (NHPs) are often the species of choice when extensive immunogenicity assessments such as total anti-capsid antibody assay, pre-existing and treatment induced anti-capsid NAb assay, and total anti-transgene product antibody assay are conducted. The immunogenicity evaluation and its general rationale for rAAV-based gene therapy in nonclinical models are listed in Table IV. The immunogenicity evaluation for an underlying disease indication may vary depending on the ROA and the extent of reliance on tissue cross-correction from the transduced tissues/cells. In the latter case, the impact of ADAs on target tissue uptake of a secreted protein in the systemic circulation needs to be addressed because it can compromise therapeutic exposure and efficacy. It is generally recognized and consistent with our experience at Takeda that local administration of rAAV is more immune privileged than IV administration (53). However, rAAV vector can be cleared into systemic circulation after local delivery such as intrathecal or intravitreal administration, and thus the risk of systemic immunological response should be assessed.

## Projection of First-in-Human (FIH) Dose and Response for rAAV-BASED GENE THERAPY

For gene therapy in general, both safety and efficacy aspects should be considered when selecting the FIH dosing regimen, even though the safety readout remains the primary end point for phase 1 or phase 1/2 clinical trials. The potential to demonstrate efficacy in a FIH study is particularly important for gene therapy, which is typically dosed as a single administration. This is especially true if the dosing procedure may pose substantial safety risks and/or if the gene therapy product is administered to pediatric populations (5, 54).

Although gene therapy is viewed as a new modality, the traditional empirical dose scaling (Eq. (1)) is still an applicable method to project clinical doses from nonclinical species. The dose in animals (in total vector genomes [vg] or capsid particles [cp]) is defined as either a no-observed-adverse-effect level (NOAEL) dose or pharmacologically active dose (PAD). It should be noted that components in Eq. (1) (i.e., species, AAV dose, and the adjustment factors) are often different between safety and pharmacology projections. Therefore, rational comparisons between the PAD and NOAEL doses should be made to justify the margin of safety (MOS). To project the human dose, the scaling factor is determined by the relevant difference in physiology and ROA: the total dose in vg or cp can be scaled by body weight if the gene therapy drug product is administered intravenously, or by organ weight or volume if it is injected into an organ with a well-defined structural boundary (Eq. (2)). In clinical studies, the IV administration is generally dosed by body weight (vg/kg), and local injection by fixed dose (vg/subject) or fixed concentration (vg/mL/subject). Lastly, we also propose using the activity factor as a combined metric that denotes expected interspecies difference in transduction and transgene expression between animal models and human.

1 $${\mathrm{Dose}}_{\mathrm{Human}}={\mathrm{Dose}}_{\mathrm{Animal}}\bullet \left(\mathrm{Scaling}\ \mathrm{factor}\right)\bullet \left(\mathrm{Activity}\ \mathrm{factor}\right)$$

2 $$\mathrm{Scaling}\ \mathrm{factor}=\frac{{\left(\mathrm{Body}\ \mathrm{or}\ \mathrm{or}\mathrm{gan}\ \mathrm{metric}\right)}_{\mathrm{Human}}}{{\left(\mathrm{Body}\ \mathrm{or}\ \mathrm{or}\mathrm{gan}\ \mathrm{metric}\right)}_{\mathrm{Animal}}}$$

One example for leveraging nonclinical information and limited clinical data for phase 1/2 dose escalation comes from valoctocogene roxaparvovec. In its nonclinical data package preceding the FIH study, the modality demonstrated sustained FVIII transgene expression in mouse models across a wide dose range (2 × 10^12^ to 2 × 10^14^vg/kg; Table V) and was reasonably safe at the top dose (19). Limited NHP data also showed therapeutic levels of FVIII at peak (Table V). As the vector is IV-administered, dose scaling was simply body weight-based. The FIH dose of 6 × 10^12^vg/kg was chosen presumably due to lack of safety data on AAV5 in human at the time of the study, as well as the dose level demonstrating potentially therapeutic FVIII level in mice (Table V). As the only human subject at this dose level did not show detectable FVIII, the dose was escalated to 2 × 10^13^ vg/kg, and again to 6 × 10^13^vg/kg after the only subject in the middle dose cohort showed sub-therapeutic level of FVIII (Table V) (55).

**Fig. 2:**
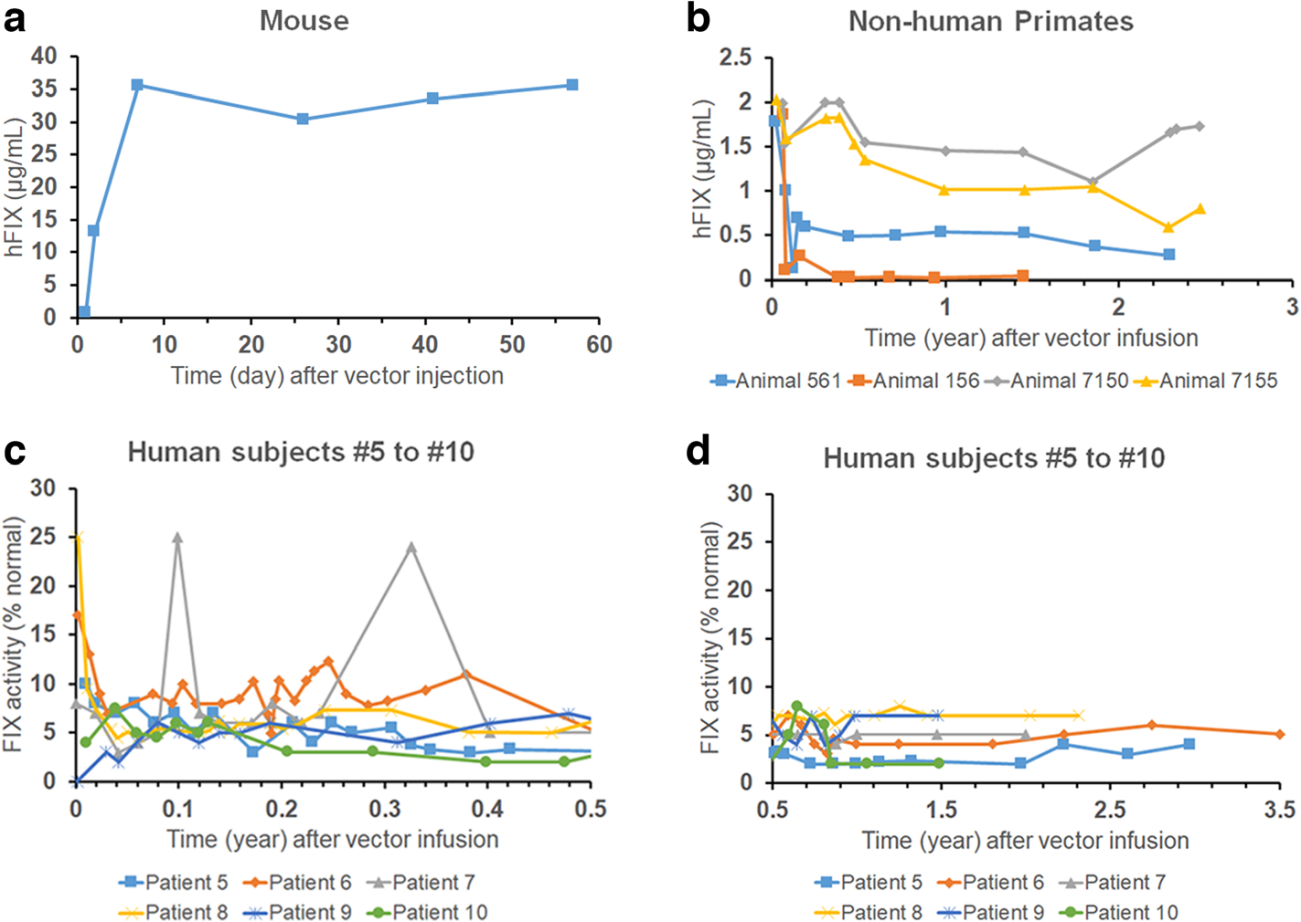
Human factor IX (FIX) levels after intravenous administration of 2 × 10^12^vg/kg scAAV2/8-LP1-hFIXco vector into mouse (**a**), non-human primates (**b**), or human subjects (**c** and **d**). Panel (**c**) shows human FIX activity up to half a year post vector infusion, and (**d**) displays hFIX activity ranging from 0.5 up to 3.5 years post vector infusion

Recently, the PAD-based human dose projection approach has been bolstered by the release of transgene expression data from clinical programs and reverse translation with existing nonclinical data. Among different types of AAV programs, the most complete component of the data package is that for liver-based gene therapy with a secreted transgene product, such as those for hemophilia, and it provides a comprehensive overview of interspecies difference in the overall response to rAAV. One well-known example is scAAV2/8-LP1-hFIXco, which demonstrated tropism towards the liver and expressed a secreted human FIX protein (56). The peak circulating FIX levels in multiple species served as an indicator for transduction efficiency of the hepatocytes and showed a clear reduction from mouse-to-NHP (20-fold decrease) and NHP-to-human (3-fold decrease; Figure 2 and Table V). In addition, in several patients, a peak-and-trough pattern in circulating FIX levels was evident, which initially dropped by 60 to 70% from peak FIX levels within the first few months after vector administration, but eventually stabilized for several years (Figure 2(c), (d), and Table V). In other investigational liver-based rAAVs for hemophilia, varying NHP-to-human FVIII or FIX ratios were observed when dose-normalized peak FVIII or FIX levels were compared between NHP and human data (Table VI), which provide a range of activity factors for use in Eq. (1) if human dose is to be scaled from NHP. Of note, these phenomena observed in liver-based gene therapy may not apply to other target tissues such as the brain, heart, and muscle, and more comparative data would be needed to confirm these results. Collectively, data on interspecies difference from reverse translation exercises should inform the forward translation strategies such as using Eq. (1) for human dose projection.

**Table V:** Levels of Human Factor IX (FIX) in Different Species Following Intravenous Administration of Two Liver-Based AAVs

	Mouse	Non-human primate	Human
Valoctocogene roxaparvovec			
Characteristics	FVIII/RAG2 double knockout (DKO) or RAG2 knockout (8-9 weeks old, male) (19)	Cynomolgus (~ 4 years old, male) (19)	Adult male with severe hemophilia A (55)
FVIII in plasma, Mean	RAG2 KO, day 35: 8.8, 64 and 273 ng/mL at 6×10^12^, 2×10^13^ and 6×10^13^ vg/kg DKO, day 56: 6.9, 46.8 and 355 ng/mL at 2×10^12^, 2×10^13^and 2×10^14^vg/kg; day 91: 79 and 322 ng/mL at 2×10^13^and 6×10^13^ vg/kg	13.8 ng/mL (peak) at 1.0×10^13^ vg/kg 43.5 ng/mL (peak) at 3.6×10^13^ vg/kg	Not detectable at 6×10^12^ vg/kg 2 IU/dL at 2×10^13^ vg/kg >10 IU/dL at 6×10^13^ vg/kg
scAAV2/8-LP1-hFIXco			
Characteristics	C57BL/6, male, 6-8-weeks old (56)	Rhesus macaques, male (73, 74)	Adult male with severe hemophilia B (73, 75)
Peak FIX in plasma, mean	36 μg/mL (week 1 through 8)	1.8 μg/mL (week 1)	11 IU/dL (~ 0.55 μg/mL)
FIX level 4 months to ~2 years, mean	N/A	0.85 μg/mL	5.1 IU/dL (~ 0.25 μg/mL)
FIX level at 8 years, mean	N/A	N/A	5.1 IU/dL (~ 0.25 μg/mL)

Upper table: Levels of human factor VIII (FVIII) in different species after intravenous administration of valoctocogene roxaparvovec. For nonclinical data, only showing those presumably generated prior to the first-in-human study. *N* = 6–10 per dose group for mouse, 2 for NHP, 1 for low and middle dose in human

Lower table: Levels of human factor IX (FIX) in different species at three time periods after intravenous administration of 2 × 10^12^vg/kg scAAV2/8-LP1-hFIXco vector, with an AAV8 capsid and self-complementary genome containing a codon-optimized human factor IX sequence. A dose adjustment of 10× due to updated titer method was applied to mouse and NHP (76). *N* = 5–8 for mouse, 4 for NHP, 6 for human. For FIX activity, assume 1 IU is equivalent to 5 μg FIX (73)

**Table VI:** Comparison of Peak Levels (C_max_) and Peak Time (T_max_) of Circulating Transgene Product from the Same rAAV Vector Administered to Non-human Primates (NHP) and Human

Vector (capsid, DNA conformation)	Peak transgene product levels and time point in NHP	Peak transgene product levels and time point in human	NHP-to-human peak ratio, dose-adjusted	Notes
Valoctocogene roxaparvovec (AAV5, ssDNA)	35 ng/mL at 6E13 vg/kg, week 4 to 5; 7/10 animals developed anti-hFVIII ADAs	Geometric mean of 92 IU/dL at 6E13 vg/kg; range: 14 – 211 IU/dL, T_max_21-46 weeks	0.38	NHP: N = 10; human: N = 7 (20, 55)
UniQure AMT-060 (AAV5, ssDNA)	13% normal hFIX at 5E12 vg/kg, week 1; one animal developed anti-hFIX ADAs	Mean level of 7.5% normal hFIX at 5E12 vg/kg; mean level of 10% normal hFIX at 2E13 vg/kg. T_max_12-28 weeks	3.5 (average of 1.7 and 5.2)	NHP: N = 3, levels adjusted with baseline; human: N = 5 (77, 78)
Etranacogene dezaparvovec (AAV5, ssDNA)	220% normal hFIX at 2.5E13 vg/kg, week 8; all animals developed anti-hFIX ADAs 400% normal hFIX at 9E13 vg/kg, week 6; one animal developed anti-hFIX ADAs	Mean level of 48.8% normal hFIX at 2E13 vg/kg, T_max_ later than 20 weeks	2.7 (average of 3.61 and 1.82)	NHP and human: N = 3 at each dose level (62, 77)
Giroctocogene fitelparvovec (AAV6, ssDNA)	228% normal hFVIII at 6E12 vg/kg, T_max_ at week 1 or 2	Mean of 119% normal hFVIII at 3E13 vg/kg, T_max_11-20 weeks	9.6	NHP: N = 3; human: N = 5 at each dose level (79)
Ultragenyx DTX-201 (AAVhu.37, ssDNA)	22.8% normal hFVIII at 1.2E13 vg/kg, T_max_ at week 2 to 4, or later than week 24 (one animal)	3.5% – 72% normal hFVIII across 5E12 – 2E13 vg/kg, T_max_4-37 weeks	1.86	NHP: N = 5; human: N = 2 at each of the three dose levels (80, 81)

Long-term NHP data is less useful due to sharp drop in transgene product levels (anti-transgene product neutralizing antibodies being a potential culprit). *FVIII*, Factor VIII; *FIX*, Factor IX; *vg*, vector genome(s); *ssDNA*, single-stranded DNA. The dose-adjusted NHP-to-human peak ratio serves as an indicator of the “closeness” of NHP to human translation and is calculated as: $\mathrm{NHP}\ \mathrm{to}\ \mathrm{human}\ \mathrm{ratio}=\frac{{\left(\mathrm{Transgene}\ \mathrm{product}\right)}_{\mathrm{NHP}}/{\mathrm{Dose}}_{\mathrm{NHP}}}{{\left(\mathrm{Transgene}\ \mathrm{product}\right)}_{\mathrm{Human}}/{\mathrm{Dose}}_{\mathrm{Human}}}$

Despite advances in understanding translational aspects of rAAV-based gene therapy, numerous gaps remain for projecting a clinically safe and efficacious dose and the durability of response. One challenge is the impact of T cell-mediated immune response against AAV-transduced host cells and the high inter-subject variability on the extent of decline from the peak level of transgene product. Another is predicting the long-term rate of decline for transgene product levels in target tissues, for which multiple mechanisms have been proposed, including growth and turnover of host cells, cell stress due to unfolded protein response, and vector-specific properties (capsid, transgene, manufacturing platform, and process) (57). However, it is yet unclear whether any causal relationships exist between these potential covariates and the trend (either decline or stabilization) of the transgene product level beyond one-year post-dose (57). Therefore, to project the duration of exposure and persistence of response for phase 1/2 trial designs, scenario planning on short- and long-term decline in transgene product levels is recommended, which may be combined with physiologically based or semi-mechanistic modeling and simulation (58) that incorporate transduction, transgene expression, and the potential impact of immune response on PK/PD (59, 60).

## Learning Opportunities

The regulatory approvals for Luxturna® and Zolgensma® are the result of several decades of technological innovations. Several other viral gene therapies undergoing clinical trials are showing promise of functional cure for the investigated diseases (59, 61, 62). However, challenges remain in the development of rAAV-based gene therapy, including persistence of efficacy, immunogenicity, the ability to re-dose, and manufacturing scalability. The lessons learned from approved therapies and ongoing clinical trials can be leveraged for future opportunities to close these gaps.

Biodistribution in most cases has been investigated primarily at the macro level of the tissues of interest. However, less well understood are the micro-level distribution of the vector into the tissue or cells of a given organ including preferential entry of the rAAV into different cell types within the organs, rAAV receptor binding kinetics, intracellular trafficking, uncoating, and mechanisms of episomal loss. Furthermore, the mechanisms responsible for the bystander effect associated with the movement of the transgene protein from transduced to non-transduced cells have not been fully elucidated (63). In addition, factors that affect transduction efficiency include the fraction of transduced target cells, cellular turnover rate, the age of cells, and metabolic state of the cells. Understanding the differences between these micro processes in both animals and humans can offer opportunities to further increase the success in meaningful clinical benefits. These learnings have the potential to further improve the accuracy of FIH efficacious dose projection, where recently more mechanistic approaches are being explored (58, 60, 64).

Research from both academia and industry has been conducted to address the noticeable challenges associated with the humoral and cell-mediated immune response (65–67). Epitope mapping of the capsid peptide or transgene sequence used to identify the immunogenic spot have become part of the routine screening process in the early discovery phase. During the screening phase, it is critical to evaluate patient-derived cells because this evaluation may improve predictability of the investigational product in the clinical setting. Following administration of rAAV, the post-dose immunogenicity assessment is typically focused on the peripheral blood compartment. However, expanding the immunogenicity evaluation into tissues such as the liver and spleen in terms of lymphocyte infiltration and immune profiling of the cellular immunogenic response may help further explain the loss of efficacy or decrease in transgene expression. Tissue-originating immune response can also be a factor in eliminating the transduced target tissue/cells where peripheral blood levels can be low or undetectable (44). Since human transgene products could induce an immunogenic effect in animal models, the use of species-specific transgene may be valuable from an immunogenicity standpoint. While the intensity and the type of immunogenicity may differ between animal models and humans, it is important to recognize that the consequence of an immune response might still occur in the clinic. For example, an NHP model was used to evaluate application of intensive T cell-directed immunosuppressant combined with AAV-mediated transfer of the human FIX gene, and the timing of T cell-directed immunosuppressant regimen was found to be critical in determining transgene-product immunogenicity or tolerance (68). As this field evolves, it will be increasingly possible to better determine the appropriate time to apply the immunosuppressant regimen to mitigate loss of transgene expression.

## Future Outlook

Before the approval of the next wave of rAAV-based gene therapies, research on this platform will continue to address the impact of immunogenicity on safety and efficacy, low transduction efficiency, re-dosing potential, and durability of efficacy. The recent exponential growth in rAAV-based clinical trials speaks volumes to this modality’s promise for improving human health, which will require efforts from multidisciplinary functions. Building on the existing roles, capabilities, and knowledge within the DMPK function, more strategic and novel approaches to efficiently assess the delivery of rAAV-based gene therapy are forthcoming. More importantly, enabling the translation from bench to bedside is where DMPK can further contribute. These contributions can include but are not limited to (a) *in vitro* experiments to understand tissue and cellular uptake of the rAAV capsid, (b) the adaptation of intracellular trafficking and transduction of transgene techniques in relevant animal and human cells, (c) an enhanced understanding on the fraction of bio-production tissues/organs and cell type population that need to be transduced, even for commonly targeted organs like the liver, (d) a better selection of appropriate animal models for pharmacological and toxicological assessment, and (e) implementation of innovative imaging techniques to enable assessment of the transduction efficiency in tissues. In summary, the knowledge gained from these studies helps to correlate the findings from the animal model to humans, addresses PK/PD translation gaps, and further improves model predictability for the FIH dose.

Compared with traditional therapeutic modalities, rAAV-based gene therapy is still in the early stage of development and medical application. While rAAV-based gene therapies are already being developed in the clinic and used in real world settings, the learnings can be applied to the next generation of gene therapies. Thus, the DMPK function along with other multidisciplinary functions needs to adapt to this rapidly evolving modality, be innovative when advancing the drug product to clinical settings, and obtain a better clinical translation to ensure safe and efficacious treatments for diseases with an unmet medical need.
